# Innovative diagnostic techniques and their clinical implications in food allergy: current clinical practice and future perspectives

**DOI:** 10.3389/falgy.2026.1835353

**Published:** 2026-06-10

**Authors:** Carlo Maria Rossi, Stefania Merli, Linda Borgonovo, Michelle Giardina, Monarita Yacoub, Baoran Yang, Francesca Losa, Fabio Lodi Rizzini, Serena Nannipieri, Marta Piantanida, Patrizia Pignatti, Donatella Preziosi, Federica Rivolta, Andrea Toniato, Serena Traversi, Alessandro Vrenna, Martina Votto, Antonio Di Sabatino, Vincenzo Patella

**Affiliations:** 1 First Department of Internal Medicine, Fondazione IRCCS Policlinico San Matteo, Pavia, Italy; 2 Allergology and Immunology Unit, ASST GOM Niguarda, Milan, Italy; 3 Dipartimento Scienze Cliniche e Sperimentali (DSCS), Scuola di Specializzazione in Allergologia e Immunologia Clinica, Università Studi di Brescia - SSvD Allergologia, Spedali Civili, Brescia, Italy; 4 Department of Internal Medicine, IRCCS San Raffaele Scientific Institute, Multidisciplinary Advanced Center Asthma, Food and Drug Allergy, Vita-Salute San Raffaele University. Ospedale San Raffaele, Milan, Italy; 5 SC Allergologia, Reumatologia e Immunologia Clinica, Italy; 6 Allergy and Immunology Unit, Istituti Clinici Scientifici Maugeri, IRCCS, Pavia, Italy; 7 Allergy Unit, Division of Pulmonology, Sant'Anna Hospital, Como, Italy; 8 Department of Internal Medicine, Fondazione IRCCS Cà Granda Ospedale Maggiore Policlinico, Milan, Italy; 9 Allergy Unit, Humanitas Gavazzeni, Bergamo, Italy; 10 Maternal and Child Health Department, Azienda Sanitaria Locale of Benevento, Mantova, Italy; 11 First Department of Internal Medicine, Department of Medicine and Medical Therapeutics, Fondazione IRCCS Policlinico San Matteo, Università di Pavia, Pavia, Mantova, Italy; 12 Department of Internal Medicine and Division of Allergy, Clinical Immunology Battipaglia Santa Maria Della Speranza Hospital, Salerno, Italy; 13 Post Graduate School in Allergy and Clinical Immunology, School of Medicine, University of Naples Federico II, Naples, Italy

**Keywords:** artificial intelligence, basophil activation test, component-resolved diagnostics, epitope-specific igE, igG4/IgEG ratio, mast cell activation test, multi-omics

## Abstract

Food allergy (FA) is an immune-mediated adverse reaction to food components (mainly proteins) and represents an increasing global health problem, affecting millions of individuals and imposing substantial clinical, psychosocial, and economic burdens. Accurate diagnosis is essential to prevent life-threatening reactions while avoiding unnecessary dietary restrictions and impaired quality of life. Current diagnostic approaches rely on clinical history, skin prick tests (SPT), measurement of serum allergen-specific IgE (sIgE), and oral food challenges (OFC). However, SPT and sIgE are highly sensitive but lack specificity, frequently identifying clinically irrelevant sensitization, whereas OFC remains the diagnostic gold standard despite being resource-demanding and carrying a substantial risk of systemic reactions. In recent years, several innovative diagnostic approaches have emerged with the aim of improving diagnostic accuracy and reducing reliance on OFC. Component-resolved diagnostics (CRD) enable detailed characterization of molecular sensitization profiles, supporting improved risk stratification and identification of clinically relevant cross-reactivity patterns. Functional cellular assays, including the basophil activation test (BAT) and the mast cell activation test (MAT), offer direct assessment of IgE-mediated effector cell responses and have demonstrated higher diagnostic specificity compared with conventional testing. Epitope-specific IgE profiling and the allergen-specific IgG4/IgE ratio may additionally contribute to a better understanding of disease phenotype and evolution. Furthermore, advances in multi-omics technologies combined with artificial intelligence and machine learning are creating new opportunities for biomarker discovery and predictive modelling in FA. This narrative review summarizes current innovative diagnostic techniques in food allergy, discussing their clinical applications, limitations, and future directions toward more precise and personalized diagnostic approaches.

## 1 Introduction

Food allergy (FA) is an immune-mediated adverse reaction to otherwise harmless food components, mainly proteins, and represents a growing global health problem (1, 2). It affects millions of individuals worldwide and is associated with substantial healthcare costs, dietary restrictions, psychological stress, and impaired quality of life for patients and their caregivers. Epidemiological studies suggest that FA affects up to 10% of the population in some regions, with an estimated prevalence of approximately 1 in 10 adults and 1 in 12 children in the United States, while European data indicate a lifetime self-reported prevalence of around 6% (3). Although prevalence estimates vary due to methodological differences, there is consistent evidence of an increasing burden over time, including a rise in food-induced anaphylaxis and FA-related hospitalizations (4, 5). The societal impact of FA extends beyond medical costs, encompassing school and work absenteeism, anxiety related to accidental exposures, and reduced participation in social activities (1).

Accurate diagnosis of FA is essential to prevent both underdiagnosis, which may expose patients to life-threatening reactions, and overdiagnosis, which can lead to unnecessary dietary avoidance and nutritional deficiencies. Current diagnostic pathways rely on a combination of clinical history, and first level tests, such as skin prick tests (SPT), measurement of serum specific IgE (sIgE) to allergen extracts or components, and oral food challenges (OFC). SPT and sIgE testing are widely available and highly sensitive, but suffer from several limitations, as summarised in Table 1 and the subsequent paragraph, the most important being variable and often reduced specificity.

**Table 1:** Advantages and disadvantages of current techniques in the diagnosis of food allergy.

Test	PRO	CONS	Cost	Availability	References
Skin Prick test	- High sensitivity and high NPV - Feasible - Rapid results - Very low adverse reaction rate - Allows testing with fresh foods	- *In vivo* - Low specificity, low PPV - Interference from antihistamines (anti-H1) - Requires healthy skin, eczema-free skin - Does not discriminate between sensitization and allergy **SPT with commercial extracts:** - Limited availability of whole extracts - Lack of standardisation, variable protein concentrations **Prick to prick testing:** - Limited availability for seasonal foods	*+*	High	(10, 11, 21)
sIgE for extracts	- High sensitivity, high NPV - *In vitro* assay - Good performance for some allergens, particularly those with limited available CRD - Quantitative, allows to measure exact quantity of allergen-specific IgE - Standardized methodology	- Low specificity and low PPV - No/limited discrimination between sensitization and allergy - Poor discrimination between true sensitization and cross-reactivity - Limited ability to predict reaction severity - Limited availability for whole extracts	*+*	High	(10, 11, 21)
Oral Food Challenge	- Gold standard - Discrimination between sensitization and allergy - Tolerance threshold determination	- *In vivo* test - Risk of adverse reactions - Requires specialized personnel - Complex interpretation for subjective symptoms - Limited availability for seasonal foods - Limited standardized protocols	*++++*	Low/Medium	(10, 11, 14, 21)

Anti-H1, antihistamines drugs blocking H1 receptor; CCD, cross-reactive carbohydrate determinants; CRD, component- resolved diagnostic; NPV, negative predictive value, OFC, oral food challenge; PPV, positive predictive value; sIgE, specific immunoglobulin E, SPT, skin prick tests;+low; ++ moderate; +++ high; ++++ very high.

These limitations provided a strong rationale for the development of innovative diagnostic approaches in FA. These aim to deliver accurate, minimally invasive, and clinically meaningful results while reducing reliance on OFC. In fact, OFC remains the gold standard for confirming clinical reactivity, despite its risks and logistical complexity. While OFC has been shown to improve quality of life by clarifying diagnosis and reducing unwarranted food avoidance, it carries a non-negligible risk of systemic reactions and requires specialized settings and trained personnel (6–9).

Component-resolved diagnostics (CRD), also referred to as molecular allergy testing, is a technique that detects sIgE antibodies to purified native and recombinant allergens, and can improve specificity and provide valuable information on cross-reactivity, persistence of allergy, and risk stratification, yet they still do not fully replace the need for OFC in many patients (10–14).

Cellular assays are functional tests; among them the basophil activation test (BAT) has demonstrated higher diagnostic accuracy than conventional tests and correlates with reaction severity, particularly in peanut, tree nut, and sesame allergy. The BAT has recently been recommended in European guidelines as a confirmatory test for IgE-mediated FA, although its availability remains limited to specialized centres (15–20).

Moreover, among functional assays, the mast cell activation test (MAT) has recently been shown to provide informative results in cases with non-responsive basophils in whom BAT cannot be used (21). It has shown robust performance to confirm peanut allergy or Bet v1-associated-FA (22, 23). The BAT has further been suggested as a potentially useful tool for monitoring oral and sublingual immunotherapy in patients with FA but data are discordant, so further studies are needed (24–28).

In parallel, research-based approaches including epitope-specific IgE profiling and IgG4/IgE ratios offer promising insights into disease phenotypes and prognosis (29, 30). Furthermore, integration of novel biological samples with omics technologies, combined with machine learning and artificial intelligence, holds the potential to identify robust biomarkers and enable molecular and functional endotyping of FA. Collectively, these techniques may improve diagnostic precision of FA, while reducing unnecessary OFC and supporting more informed clinical decision-making. By enabling a more accurate distinction between true FA and asymptomatic sensitization, innovative diagnostic strategies have the potential to substantially reduce the clinical and social burden of FA (31).

**Figure 1:**
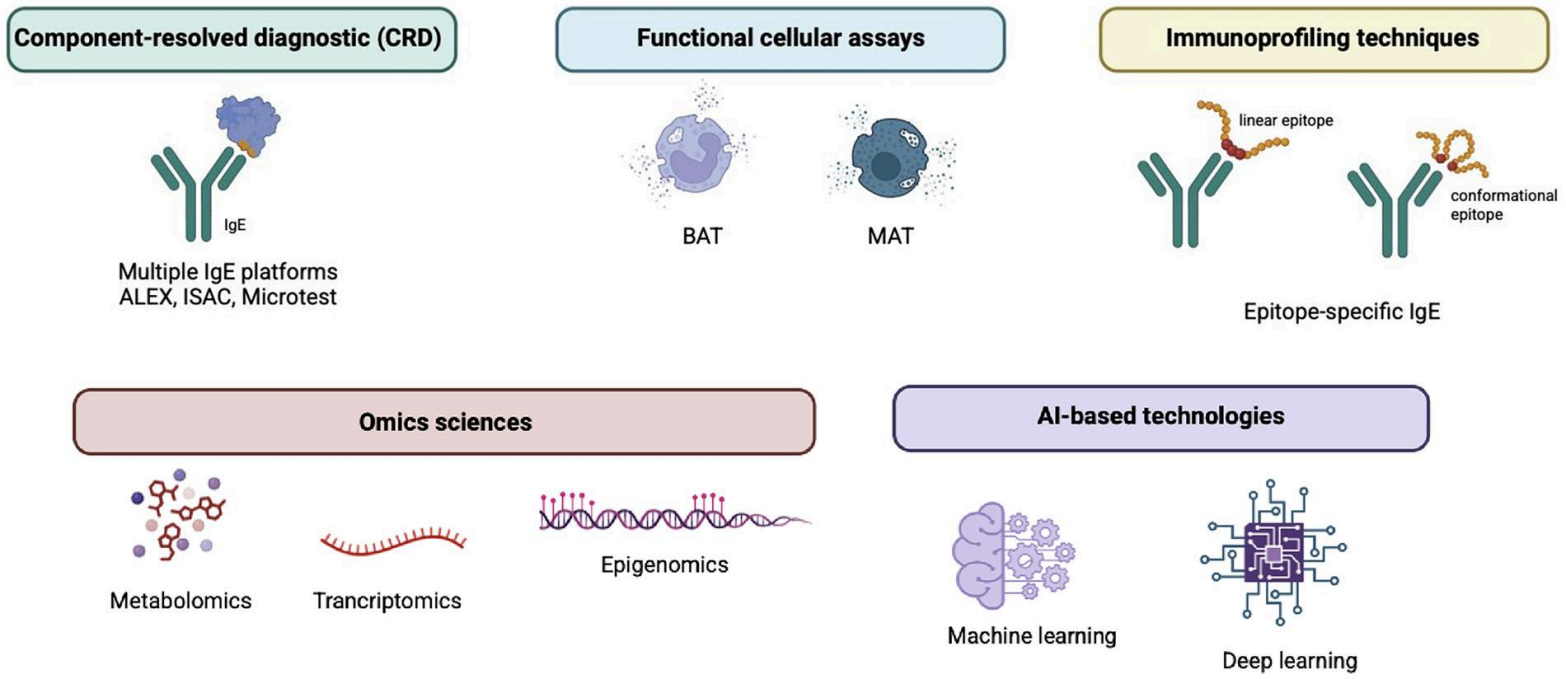
The figure shows innovative diagnostic techniques. They include component resolved diagnostics (CRD), functional cellular assays, such as the basophil activation test (BAT) and the mast cell activation test (MAT); immune-profiling techniques, such as epitope analysis, as illustrated in the picture by the analysis of conformational and linear epitopes, the ratio of IgG4 to IgE, not depicted, omics technologies (e.g., metabolomics, trascriptomics, epigenomics) and artificial intelligence (AI)-based methods including machine learning and deep learning. *Created with Biorender.com.*

In this context, innovative diagnostic approaches in FA can be broadly grouped into molecular diagnostics (CRD), functional cellular assays (the BAT and the MAT), advanced immune-profiling techniques, omics-based biomarker discovery, and artificial intelligence (AI)–driven predictive models, as summarised in the Figure 1.

This narrative review summarises the use of innovative techniques in the diagnosis and management of FA, highlighting their clinical implications, existing challenges and limitations, and potential future directions.

This narrative review was conducted through a literature search of PubMed, Web of Science, and Scopus for English-language articles published up to October 2025. Priority was given to position papers, guidelines, and consensus documents, supplemented by relevant original research and reviews. Evidence was synthesized narratively, considering study quality and relevance, without formal scoring or risk of bias assessment. Given the rapid evolution of the field and the heterogeneity of study designs, particularly in emerging areas such as AI and multi-omics approaches, findings should be interpreted with caution regarding potential publication bias and early overestimation of clinical applicability.

## 2 Limitations of the traditional diagnostic tools

Clinical management of FA currently relies on a combination of history, sensitization testing, and, when necessary, OFC, which remains the diagnostic gold standard. Diagnosing FA is often challenging and limited as first-line tests, including SPT and sIgE, cannot reliably distinguish immunological sensitization and clinically relevant allergy. The high sensitivity but relatively low specificity of these tests frequently results in false-positive findings and overdiagnosis, particularly in populations with a high background rate of atopy (10, 21). This limitation is further compounded by the lack of universally applicable diagnostic cutoff values, as threshold levels vary widely depending on the specific allergen, patient age, geographic region, and underlying population characteristics.

Commercial extracts used in SPT may differ in protein content, stability, and representation of clinically relevant epitopes, potentially leading to inconsistent or misleading results. Moreover, SPT cannot be performed in case of recent antihistamine use or in patients with atopic dermatitis/dermographism and, although very rarely, can lead to systemic allergic reactions (10, 21).

sIgE are more expensive than SPT and can be influenced by nonspecific cross-reactive proteins. This is especially relevant in patients with pollen allergies, where sensitization to panallergens, such profilins or pathogenesis-related (PR)-10 proteins lead to positive test results for a wide range of plant-derived foods without corresponding clinical reactivity. As a result, both SPT and sIgE testing may overestimate the number of true food allergies.

Although OFC is the gold standard for FA diagnosis, it also has significant limitations, including interpretation bias and psychogenic influences from patients (32). Given the risk of potentially life-threatening reactions, OFC requires qualified infrastructure and healthcare personnel capable of recognizing and treating allergic reactions. Meeting high demand can be difficult, leading to long waitlists, even at specialized centres, which delay the timely introduction of foods into diets. Some families are anxious about undergoing OFC and cannot stop taking allergy medications. The patient should be well and have their atopic conditions under control, such as asthma and atopic dermatitis, leading to frequent cancellations (21). Given these constraints, clinicians typically reserve OFC for cases in which the diagnosis remains inconclusive after an allergy-focused clinical history and diagnostic test (SPT and sIgE measurement). However, these tools do not reliably predict disease persistence, reaction severity, or response to emerging therapies such as oral immunotherapy (OIT) (21, 33).

## 3 Innovative diagnostic tools

### 3.1 Component-resolved diagnostic

CRD is a molecular diagnostic approach that uses purified native or recombinant allergens to detect the IgE antibodies against individual allergenic proteins. In the FA diagnostic this technique enables detailed characterisation of the molecular sensitization profile, thereby improving risk assessment, prediction of clinical severity, and identification of clinically relevant cross-reactivity (34). Highly standardized and automated analytical systems provide accurate, reproducible, and quantitative measurements of sIgE levels against individual molecular components, such as those obtained with ImmunoCAP (Thermo Fisher Scientific). Additionally, multiplex platforms for IgE detection, including ISAC (Thermo Fisher Scientific) and ALEX (Allergy Explorer, Macroarray Diagnostics) are currently available (35).

CRD is generally accessible in secondary care settings, while platform-based assays remain confined to tertiary-level centrers.

Of note, the CRD can discriminate true sensitization to clinically relevant allergens from cross-reactivity due to homologous proteins present in botanically unrelated foods or pollens. For example, in peanut allergy, IgE reactivity to Ara h 2 and Ara h 6 are strongly associated with genuine clinical allergy and an increased risk of severe reactions, whereas sensitization to Ara h 8, a Bet v 1 homologous belonging to the PR-10 allergy family, is more commonly associated with pollen-food allergy syndrome, also known as oral allergy syndrome (OAS) (36, 37).

Consequently, the European Academy of Allergy and Clinical Immunology (EAACI) and American Academy of Allergy, Asthma, and Immunology (AAAAI) recognize CRD as a promising adjunctive diagnostic tool; however, they do not recommend its use for random screening to assess IgE sensitisation (13, 38).

Multiplex allergen microarrays represent advanced *in vitro* diagnostic tools that play an increasingly important role in the diagnosis and management of FA. These assays allow the simultaneous detection of multiple allergen-specific IgE antibodies against a broad panel of allergen extracts, purified molecular components, and selected epitopes, using a very small serum volume (approximately 20–50 µL), which is particularly advantageous in paediatric patients (39, 40). The technology is based on solid-phase platforms, including microarrays or macroarrays (glass slides or chips) onto which multiple allergens are immobilized. It enables high-throughput analysis and detailed molecular sensitization profiling through CRD, which is not feasible with traditional extract-based singleplex assays (41), since these would require a huge amount of serum. In addition, multiplex testing may reduce both time and costs compared with multiple singleplex measurements. Further innovations include the use of recombinant allergens to improve specificity, the development of reverse-phase arrays, enabling high-throughput, multiplex analysis of IgE responses using minimal sample volumes and photocleavage-based affinity purification (PC-PURE), which ensures high purity and structural integrity of allergenic molecules—and the application of big-data analytics to optimize allergen panels for global clinical relevance (42, 43).

Several commercial multiplex IgE platforms are available, including ImmunoCAP ISAC (Thermo Fisher Scientific, Sweden), ALEX2 (Allergy Explorer, MacroArray Diagnostics, Vienna, Austria), and Microtest (Microtest Diagnostics, London). ISAC is the most widely used and best characterized microtest system, currently comprising 112 allergen components from more than 50 allergen sources, with semiquantitative results reported in ISU units (44). ALEX includes a broader panel with 157 allergen extracts and 125 molecular components, provides quantitative results expressed in kUA/L, incorporates total IgE measurement, and uses a cross-reactive carbohydrate determinant (CCD) inhibitor to reduce false-positive results due to this component. To enhance global exposome coverage and improve test sensitivity, the allergen panel was upgraded from ALEX2 to ALEX3. ALEX3 includes 218 allergens and 82 extracts, with 52 new allergens added. The use of a large dataset from ALEX2 test results demonstrates a data-driven approach to refining the diagnostic IgE-macroarray. Overall, ALEX3 provides broader coverage of global allergen exposure, for instance by expanding the representation of clinically relevant families, such as seed storage proteins, while removing low prevalence (<1%) allergens. It therefore represents a further step toward more precise molecular allergy diagnostics. More precisely, ALEX3 improves global coverage by incorporating allergens relevant to different geographic regions and introduces new surrogate markers for pan-allergen families, such as cyclophilins (43). Microtests offers a more limited allergen repertoire (45, 46).

Overall, multiplex microarray results generally correlate well with singleplex assays for major food allergens. However, discordant results may occur, particularly in the presence of high IgG or IgG4 levels, which can block IgE binding to a small amount of allergen present in the microchip, and negative results do not fully exclude clinical allergy (39)*.* Interpretation must always be considered within the clinical context. Multiplex allergen microarrays are helpful in patients with complex or unclear allergic symptoms, multiple sensitizations, and multiple food and/or inhalant cross-reactivities. They also assist in the management and risk stratification of FA and in the evaluation of patients before an OFC. However, they do not replace OFC, which remain the “gold standard” for the diagnosis of FA, and they require expert interpretation (47).

Table 2 summarises the limitations of the innovative techniques. More specifically, general limitations of CRD include variability in diagnostic thresholds, geographic differences in sensitisation profiles, and the need for further validation in large, well-characterised cohorts. In addition, the relatively high cost of CRD raises concerns regarding its cost-effectiveness in routine clinical practice (48). In summary, CRD aims to define the molecular sensitization profile in patients with FA, supporting a more precise diagnosis and personalized treatment plan, nevertheless its diagnostic and clinical utility is not absolute, and results must always be interpreted in the clinical context and in the light of current evidence.

**Table 2:** Advantages and disadvantages of innovative techniques in the diagnosis of food allergy.

Test	PRO	CONS	Cost	Availability	Clinical readiness and use/ setting	References
Singleplex molecular allergen diagnostic	- High specificity - *In vitro* assay - Discriminates between true sensitization and cross-reactivity - Prognostic value - Risk assessment before OFC	- Does not fully discriminate between sensitization and allergy - Limited availability for molecular component	*++*	High	Ready, secondary care	(11–13, 38)
Multiplex allergen microarrays	- Simultaneous testing of hundreds of allergen components and/or extracts - Efficient for polysensitized patients - Broad sensitization profile assessment in a single assay - Useful for epidemiological studies and complex cases	- Variable analytical performance among allergens - Some platforms show lower sensibility compared to singleplex assays - Increased risk for false-positive results in some system due to insufficient CCD inhibition	*+++*	Medium	Ready, tertiary care	(11, 39–44)
Basophil activation test (BAT)	- High sensitivity, specificity, - *In vitro* assay - Sensitive at low IgE levels - High prognostic value - Risk assessment prior to OFC - Applicable to whole allergen extracts and molecular components - -Discrimination between sensitization and allergy	- Requires specialized operators - Requires fresh blood - 10–15% of non-responders - Limited possibility for batch testing - Lack of standardized protocols - Limited availability of extracts/components	*+++*	Low	Partially validated, tertiary care in selected cases	(11, 12, 15–19, 21, 25, 28)
Mast cell Activation Test (MAT)	- High specificity and PPV - *In vitro* assay - Sensitive at low IgE levels - Virtually no ‘’non-responders’’ - Batch testing possible - Discrimination between sensitization and allergy (e.g., in peanut allergy)	- Diagnostic performance still under evaluation - Lack of standardized protocols - Requires specialized operators - Time-consuming and costly cell generation - Limited clinical validation - Limited availability of extracts/ molecular component	*++++*	Very low	Investigational, tertiary care, in selected cases	(28, 56–58)
Allergen specific IgG4/IgE ratio	- Reflects immunologic balance between allergic reactivity and tolerance - May help monitoring immunotherapy	- Cut-off values not standardised - Interpretation varies across allergens and populations - Not recommended as a standalone test	*++*	Low	Investigational, research, tertiary care	(61, 62)
Epitope-based testing	- High specificity - May predict disease persistence - Enables risk stratification	- Expensive and technically complex - Requires advanced bioinformatic analysis - Limited clinical validation	*++++*	Very low	Investigational, research, tertiary care	(64–69, 79, 80)
Multi-omics and AI-based approaches	- Enables multidimensional disease characterisation - May identify early biomarkers of FA - Potential to predict disease onset, persistence and therapy response	- Expensive and technically complex - Requires large, well-phenotyped cohorts for model training - Risk of algorithm bias and overfitting - Data privacy concerns and immature regulatory frameworks	*++++*	Very low	Investigational, research, tertiary care	(31, 71, 72, 77, 78, 81, 83, 88, 89, 93–97)

AI, artificial intelligence; BAT, basophil activation test; CCD, cross-reactive carbohydrate determinants; CRD, component- resolved diagnostic; MAT, mast cell activation test; NPV, negative predictive value; OFC, oral food challenge; PPV, positive predictive value;+low; ++ moderate; +++ high; ++++ very high.

### 3.2 Cellular assays

Cellular assays include the BAT and the mast cell activation test (MAT) and provide a measure of allergen-induced cellular activation and thus functional measure of immune reactivity beyond simple sIgE quantification, as offered by CRD.

The IgE axis is one of the principal mechanisms of basophil activation. When specific IgE antibodies recognizing a suspected allergen are bound to Fc*ε*RI receptors on the basophil membrane, subsequent exposure to the allergen can trigger basophil activation. The BAT is a functional assay used to evaluate this activation response (21). Briefly, anticoagulated blood, either with heparin or EDTA, is incubated with substances suspected of triggering allergic reactions. Upon activation, basophils undergo degranulation, during which cytoplasmic granules fuse with the cell membrane, leading to surface expression of CD63, a glycoprotein normally contained within these granules. CD203c, a lineage-specific marker of basophils, also increases in surface expression upon activation. These activation markers can be quantitatively assessed by flow cytometry.

The BAT was initially primarily used for diagnosing drug hypersensitivity reactions, but its utility has recently been demonstrated in the context of FA, so that it has been incorporated into the EAACI diagnostic algorithm for IgE-mediated FA (19), particularly in cases where SPT and sIgE results are inconsistent with the clinical history. Sensitivity and specificity of BAT vary depending on the food allergen tested, reaching up to 100% for certain foods (49). Of note, the BAT could be especially valuable in cases with low total IgE levels and negative sIgE (50).

A positive BAT, depending on the type of reaction and the implicated food allergens, may allow for a more straightforward diagnosis of FA, *i.e.*, without the need for an OFC. The BAT can also help identify patients who truly require an OFC. Indeed, in a study assessing the diagnostic role of different test for predicting a positive OFC response in children allergic to cow's milk (both baked and fresh), particularly in those under 2 years of age, the BAT was the most accurate technique as compared to skin tests and specific IgE (51). In egg-allergic patients, the BAT outperformed the other tests in both individuals partially consuming it compared to those strictly avoiding it (52). The BAT can also distinguish egg-tolerant individuals during food reintroduction (53). Moreover, it has been shown to predict successful outcomes of OIT in peanut-allergic patients when performed at baseline prior to treatment (25). Additionally, the BAT may be useful for monitoring anti-IgE therapy in patients with chronic urticaria or asthma associated with food-induced anaphylaxis (54, 55).

Limitations of the BAT include the requirement for fresh blood samples and rapid processing, within approximately four hours of blood collection, and the need for specialized operators, and flow cytometry facilities, the presence of non-responders, estimated approximately 10%–15% of patients, and the limited availability of allergen extracts and molecular components, among others, as summarised in Table 2.

The MAT is a functional *in vitro* assay that evaluates the capacity of allergen-specific IgE present in patient serum to induce mast cell activation upon allergen exposure. In this assay, cultured mast cell lines (*e.g*., LAD2) or cultured primary human mast cells are activated directly or indirectly after passive sensitisation with patient serum and subsequently stimulated with the relevant allergen; activation is quantified by measuring surface markers, such as CD63, or by assessing mediator release, such as histamine, or newly synthetised chemokines such as CCL4 OR CXCL8 (56). This approach enables direct assessment of the functional activity of allergen-specific IgE, as well as the inhibitory effects of blocking antibodies such as IgG4, and overcomes some limits of the BAT, namely the presence of non-responders, Moreover, this test overcomes other technical constraints of the BAT, including its strict time-dependency, and its potential reduction in responsiveness following systemic reactions (57). Unlike BAT, MAT allows batch testing, defined as the analysis of multiple samples in a single run, as it can be performed on stored samples, thus reducing inter-assay variability.

However, the MAT remains labour-intensive and technically demanding due to the need to obtain and culture mast cells, which are tissue-resident cells rather than blood circulating basophils, and requires specialised equipment and expertise and is therefore more expensive than BAT.

Moreover, protocol standardisation remains an unsolved issue, also with the MAT.

Comparative studies in peanut allergy have demonstrated that both BAT and MAT show high diagnostic accuracy and outperform allergen-specific IgE alone (15, 16, 58).

A direct comparison using primary mast cells derived from CD117+ progenitors showed that MAT could detect mast cell activation at allergen concentrations up to 100-fold lower than those required for BAT, indicating higher analytical sensitivity (15). Both assays were able to discriminate between clinical allergy and asymptomatic sensitization, a critical issue in FA diagnosis (57).

Collectively, however, both techniques are costly, require more analysis and standardization before they can be fully implemented as reliable routine diagnostic tools (57). More precisely, while BAT has currently a clearer diagnostic role within clinical workflows, albeit at present limited to specialised practice, MAT remains less established, only promising, and possibly complementary. Both tests are restricted to tertiary care centres with advanced diagnostic capabilities, especially for the MAT.

### 3.3 Ratio of allergen specific IgG4 to IgE

Allergen-specific IgE alone reflects sensitisation; however, it does not reliably distinguish between asymptomatic sensitisation and clinically relevant allergy. In contrast, the IgG4/IgE ratio reflects the balance between effector and blocking antibody responses, with higher ratios being associated with reduced effector cell activation and clinical tolerance (59, 60). Therefore, the ratio of allergen-specific IgG4 to IgE has emerged as a potential immunological profiling biomarker, able to improve the diagnostic interpretation of sensitisation in FA.

Several studies have shown that patients with FA typically exhibit lower allergen-specific IgG4/IgE ratios compared with tolerant/desensitized individuals, and that this ratio improves discrimination between allergic and tolerant phenotypes, particularly in peanut allergy (61, 62). Furthermore, the IgG4/IgE ratio correlates with functional assays such as the BAT and MAT and may provide additional diagnostic value when used in combination with specific IgE, skin testing, and cellular assays (60, 63). Although the IgG4/IgE ratio is not currently recommended as a standalone diagnostic test, it may serve as a useful adjunct biomarker to improve risk stratification and diagnostic accuracy, especially in patients with equivocal IgE levels or inconclusive clinical history (59–63). This test at present available in tertiary care centres.

### 3.4 Epitope-based testing

Epitope-based testing is an advanced diagnostic approach that evaluates IgE binding to specific linear epitopes within allergen protein sequences. These assays are technically robust and do not depend on protein folding; however, they reflect only part of the IgE repertoire, as conformational epitopes—often dominant in native allergens—can mask or outcompete the recognition of linear sequences, thereby impacting both biological and analytical processes. Therefore, this approach offers greater specificity but at the cost of reduced sensitivity, compared to other forms of IgE testing methods (21).

The most used platforms include peptide microarray and bead-based assays. Briefly, the allergen protein sequence is divided into overlapping peptides, which are then incubated with patient serum. IgE binding to individual epitopes is measured, and binding patterns are quantitatively analysed. The resulting output includes the number of recognized peptides, the intensity of IgE binding, and the patient-specific epitope recognition profile (21, 64, 65).

IgE reactivity to linear epitopes—such as Gal d 1 in egg allergy, Ara h 2 in peanut allergy, and casein in milk allergy—is strongly associated with clinically relevant and persistent disease, as opposed to conformational epitopes, which are more often linked to transient or less severe sensitization. Accordingly, this approach may help predict the natural history of FA by identifying broader epitope recognition and stronger binding intensity to these epitopes (67, 68). In addition, epitope-based testing enables risk stratification, as greater epitope diversity has been associated with more severe clinical phenotypes, including an increased risk of anaphylaxis. Finally, this strategy may be useful for monitoring immunotherapy, since successful treatment is typically accompanied by a reduction in IgE binding to relevant epitopes (69).

However, the main limitations of this technique include its high cost and limited availability, which currently restrict its use primarily to research settings and specialized centres (21).

### 3.5 Non-invasive methods: digital health, multi-omics approaches and AI-based integration and interpretation

The complexity of FA, shaped by genetic predisposition, environmental exposures, epithelial barrier integrity, immune regulation, and microbial interactions, requires integrative approaches capable of capturing multidimensional biological information. Multi-omics technologies combined with artificial intelligence (AI)–based analytics represent a paradigm shift in this direction. Multi-omics profiling, including genomics, transcriptomics, proteomics, and epigenomics, has been explored in various FA studies. However, the vast amount of information generated by omics science needs to be analyzed and integrated with new analytical tools, including AI and machine learning (ML) technologies (70, 71). Moreover, these approaches differ substantially in biological meaning, reproducibility, data dimensionality, cost, interpretability, and translational maturity, and should therefore be critically appraised within distinct domains rather than as a single unified paradigm.

#### 3.5.1 AI/ML-based tools

AI is increasingly recognized for its ability to transform medicine, including allergy and immunology (72). AI is a subfield of computer science that simulates human intelligence and performs tasks previously performed by humans. In the field of allergy and immunology, AI has already demonstrated many applications, including predicting asthma attack or inborn errors of immunity, phenotypes of eosinophilic gastrointestinal disorders, or identifying eczema from image data (73–76).

In FA, AI-driven models have been primarily applied to clinical prediction tasks, which should be distinguished based on their specific objectives, including onset prediction, persistence or resolution (tolerance acquisition), OFC outcome prediction, and response to OIT.

Predictive models integrating clinical history with diagnostic test results (sIgE, SPT and the BAT) with clinical history have been developed to improve diagnostic accuracy of peanut and tree nut allergy and to reduce the need for OFC (9, 32, 77).

AI approaches have been increasingly applied to predict the onset and persistence of FA in pediatric populations and have shown variable performance depending on data type and cohort characteristics. A deep learning (DL) framework based on long short-term memory (LSTM) architecture was developed to model longitudinal risk trajectories for milk, egg, and peanut allergy from birth to three years of age in cohorts from Russia, Finland, and Estonia. This model integrated gut microbiome profiles and allergen-specific sIgE measurements derived from the DIABIMMUNE dataset. The predictive performance of the DL algorithm yielded an area under the receiver operating characteristic curve (AUC-ROC) of 0.69 for clinical FA status, indicating moderate discriminative capacity (78). Within the Consortium of Food Allergy Researchers (CoFAR2) prospective cohort, investigators collected comprehensive immunological and clinical data from 293 children at high risk for FA, including peanut-specific and epitope-specific IgE and IgG4 levels, total IgE, SPT results, demographic variables, and detailed clinical histories. An ML model trained on these multidimensional parameters demonstrated robust predictive performance for peanut allergy development at four years of age, achieving AUC values ranging from 0.84 to 0.87 depending on the age at which data were incorporated (79). Similarly, in the LEAP study, clinical parameters together with biomarker profiles of IgE and IgG4 directed against 64 sequential epitopes of the Ara h 1–3 proteins were incorporated into an elastic net regression model to predict peanut allergy at five years of age, achieving predictive accuracies ranging from 64% to 83%, depending on the extent of input data included in the analysis (80). These differences underscore the importance of model calibration, dataset richness, and biological relevance of input features when interpreting predictive performance.

Taken together, these studies illustrate the potential of AI-driven predictive modeling to enhance early risk stratification and guide clinical management in FA.

Other major applications of IA in FA are the prediction of OFC outcomes and therapeutic responses to OIT (81). Initial studies combining clinical and biomarker data have demonstrated promising results (82). In a retrospective cohort of pediatric patients who completed OFCs to cooked egg, structured clinical and laboratory data were used to train an ML model incorporating demographic variables, total sIgE, and allergen sIgE levels to egg white, egg yolk, and ovomucoid. The resulting model demonstrated strong discriminative performance, achieving an AUC-ROC of 0.83 for predicting OFC success (83). Similarly, a ML–based decision tree analysis (DTA) was applied to predict the outcome of sesame OFCs and to establish the diagnosis of sesame allergy. This approach integrated demographic characteristics, detailed clinical history, sesame-specific IgE concentrations, and SPT results, yielding high diagnostic accuracy (84).

Furthermore, ML algorithms have been employed to predict sustained unresponsiveness following milk OIT in both pediatric and adult patients with cow's milk allergy. In this context, predictive modeling leveraged epitope-specific antibody binding profiles measured before and after desensitization, highlighting the potential of immune-profiling-based algorithms to inform long-term therapeutic prognosis (69).

Despite these advances, most AI models remain cohort-specific, often developed in highly selected populations using retrospective single datasets or internally split validation design (with training and validation sets performed on data drawn from the same population/institution). While these studies often report strong discriminative performance (*e.g*., AUC, sensitivity, specificity), they do not ensure generalisability across different geographic, ethnic, and clinical settings. External validation, standardization of input variables, and prospective implementation studies are essential before clinical translation.

#### 3.5.2 Multi-omics profiling

Multi-omics profiling—including genomics, epigenomics, transcriptomics, and proteomics—has been increasingly explored to characterize the biological mechanisms underlying FA (70, 71). Each layer provides distinct and complementary information but also differs in stability, interpretability, and proximity to clinical application. In parallel, AI is playing an expanding role in the analysis of -omics data, offering complementary approaches to established biostatistical methods such as epigenome-wide association studies and principal component analysis. In particular, deep learning (DL) models can capture the intricate structures within and across omics layers. This integrative capability may be especially valuable in allergy research, where intricate interactions between molecular pathways and environmental exposures are highly relevant (70, 71).

Genomic studies have identified susceptibility loci associated with immune regulation and epithelial barrier function (85). Variants in genes encoding filaggrin, T helper (Th)-2 cytokines, and HLA molecules have been consistently linked to increased risk of FA (86). Nevertheless, genetic predisposition alone accounts for only a modest proportion of disease variance, suggesting that additional regulatory layers contribute to phenotypic expression.

Epigenetic modifications, including DNA methylation and histone modifications, provide a mechanistic bridge between genetic susceptibility and environmental exposures. Early-life factors such as diet, microbial colonization, antibiotic exposure, and pollutants can influence immune programming through epigenetic remodeling. Distinct DNA methylation patterns have been observed in children with FA, particularly in genes involved in T-cell differentiation and immune regulation (87).

Transcriptomic profiling further refines disease characterization by capturing gene expression patterns in immune cells. Peripheral blood mononuclear cells and allergen-stimulated T cells from allergic individuals often show signatures consistent with Th2 polarization, including upregulation of interleukin (IL)-4, IL-5, and IL-13 pathways, along with impaired regulatory T-cell activity (89). Transcriptomic endotyping may help distinguish transient from persistent allergy and identify individuals likely to respond to immunomodulatory therapies.

Proteomic analyses complement transcriptomics by quantifying functional immune mediators and allergen-specific antibody profiles. In parallel, CRD enables precise identification of sensitization patterns to specific allergenic molecules, improving risk stratification for systemic reactions (90, 91). However, the clinical implementation of these approaches is still limited by cost, standardization, and inter-platform variability.

#### 3.5.3 Microbiome-metabolome integration

The gut microbiome is a critical ecological interface between the host and the environment. Early-life microbial diversity and composition profoundly influence immune maturation.

Metabolomics captures downstream biochemical consequences of immune and microbial interactions. Alterations in short-chain fatty acids, sphingolipids, and amino acid metabolism have been linked to impaired regulatory immune responses (92). Reduced butyrate production, often associated with decreased abundance of specific commensal bacteria, has emerged as a potential mechanistic contributor to defective oral tolerance (93). Because metabolic signatures reflect dynamic physiological states, metabolomics may serve as a sensitive indicator of disease activity and therapeutic response.

Infants who develop FA often show reduced microbial diversity, delayed colonization by beneficial taxa, and altered functional metabolic pathways (94, 95). Longitudinal studies indicate that the first thousand days of life constitute a critical window in which microbial–immune interactions shape long-term tolerance (96, 97). Integrating microbiome sequencing data with host transcriptomic and metabolomic profiles allows a systems-level understanding of these complex interactions.

## 4 Clinical implications of innovative techniques and modified traditional tests

As already discussed, inaccurate diagnosis of FA leads to unnecessary dietary restrictions, impaired quality of life, and higher healthcare costs. Conventional tests, while highly sensitive, are poorly specific, may identify clinically irrelevant sensitization, resulting in unjustified food elimination and avoidable OFCs. Such unnecessary exclusion may even promote sensitization, especially in early life (10).

FA is a complex biological phenomenon and advanced diagnostic strategies aim to improve its management, by addressing different aspects of the allergic response. These strategies may help the physician answer key clinical issues, discriminating true allergy from sensitisation, distinguishing genuine sensitisation from cross-reactivity, assessing individual risk stratification, predicting natural disease evolution, evaluating tolerance to food-processing (*e.g.*, baking), identifying or monitoring tolerance/desensitisation during/after allergen-specific immunotherapy (21). Of note, these tools should be combined sequentially within a tiered diagnostic framework to refine individual risk stratification.

More specifically, CRD have a role in estimating individual risk predicting the potential severity of reactions and by identifying clinically relevant sensitization profiles. When implemented through multiplex assays, CRD can provide a detailed map of the individual sensitization architecture through the simultaneous assessment of multiple allergenic components.

Of note, CRD has a role in potentially identifying patients at risk of systemic or severe reactions and is especially valuable in polysensitized patients. For instance, sensitization to stable plant food allergens, such as lipid transfer proteins (LTPs), which typical of the Mediterranean region, is associated with persistent allergy and increased risk of systemic reactions, including anaphylaxis (98). In peanut allergy, IgE to Ara h 2 is the most reliable molecular marker of clinically relevant allergy and is strongly associated with reaction severity. Similarly, Cor a 14 (hazelnut), Ana o 3 (cashew), and Ses i 1 (sesame) outperform whole-extract IgE in predicting true allergy and guiding dietary avoidance (1, 10). In egg allergy, IgE to Gal d 1 (ovomucoid) identifies patients less likely to tolerate heated egg, supporting tailored dietary advice. Conversely, sensitization patterns associated with pollen food syndrome—primarily involving PR-10 proteins and profilins—are typically linked to mild, localized symptoms and tolerance to cooked foods due to allergen lability. Recognition of these patterns can allow safe dietary liberalization and prevents unnecessary long-term food avoidance (99). However, the ability to predict reaction severity remains incomplete, particularly for certain allergen families such as LTP, where clinical expression is highly variable (100).

As CRD reflects IgE sensitization rather than effector cell activation, cellular assays such as the BAT may further refine risk assessment by evaluating the biological relevance of detected sensitizations and hence determine the likelihood of clinical reactivity. Accordingly, the BAT can be useful in situations of diagnostic uncertainty, such as borderline Ara h2 positivity or discordant results between clinical history and *in vitro* testing. In this instance, a negative BAT could allow to avoid an OFC, while a positive one could confirm the need for a careful OFC.

Moreover, the BAT can provide information on temporal changes in effector cell responsiveness and hence contribute to the assessment of response to immunotherapy. The MAT may be used BAT-negative or non-responder cases; however, its role in clinical practice remains investigational.

Epitope mapping provides additional interpretative depth beyond standard CRD by enabling a more precise characterization of IgE binding patterns and their potential clinical relevance and hence may be employed to identify allergy severity and persistence patterns.

IgG4/IgE ratio could be used to identify desensitisation/tolerance to allergen-specific treatments.

Finally, AI with its multi-omics approaches may prospectively contribute to a better integrated prediction at system-level, albeit its use at present largely aspirational.

However, no single biomarker at present, despite varying degrees of “maturity”, fully captures the multifaceted and dynamic nature of FA. In this instance, OFC remains central in FA management, as it serves as the definite common outcome for diagnosing clinically relevant forms and monitoring their evolution.

Moreover, given the dynamic natural history of many food allergies—particularly cow's milk, egg, wheat, and soy—periodic reassessment is crucial to confirm persistence of allergy or to support timely reintroduction of tolerated foods (10). Improving the safety and efficiency of OFCs and translating diagnostic outcomes into personalized dietary and therapeutic strategies are closely linked goals in contemporary FA care. According to a food-centred approach, diagnostic testing, including OFCs, should directly guide meaningful dietary decisions and long-term management, while being increasingly integrated with advanced diagnostic techniques for refined patient stratification, rather than serving as isolated tools for binary labelling of allergy vs. tolerance (100). In this instance, functional diagnostic tools have substantially improved pre-challenge risk stratification (51) but even the most advanced diagnostic tools ultimately require validation through controlled exposure during OFC (7).

In addition to patient selection, innovations during OFCs aim to increase early detection and improve standardized management of reactions (101). Preliminary evidence suggests that continuous physiological monitoring by means of wearable devices may detect subtle changes preceding “overt” allergic reactions, potentially enabling earlier intervention and preventing progression to more severe manifestations (102). In parallel, substantial discrepancies among commonly used anaphylaxis severity grading systems have been reported, with consequent variable use of intramuscular adrenaline during OFCs. Therefore, harmonization of severity definitions and structured team training therefore represent essential components of safer and more reproducible challenge procedures (103).

Beyond its diagnostic role, the OFC may serve a therapeutic purpose by enabling safe dietary reintroduction and guiding individualized strategies aimed at promoting immune tolerance. In this instance, contemporary strategies increasingly recognize dietary management itself as an active therapeutic tool, in which controlled exposure and structured dietary advancement may actively shape immune tolerance (100). In this context, structured milk and egg ladders, even in selected children with a history of anaphylaxis, have demonstrated feasibility and safety when carefully supervised, allowing gradual dietary expansion, improved nutritional adequacy, and meaningful quality of life improvements (104). However, successful dietary reintroduction is not solely determined by biological eligibility. Indeed, social factors influencing health, including access to allergen-free foods, nutritional counselling, school meal programs, and caregiver health knowledge, substantially influence the feasibility and sustainability of post-OFC dietary management (105). Even after negative challenges, families may delay or avoid dietary reintroduction due to residual anxiety or insufficient guidance, underscoring the importance of counselling and shared decision-making (106).

Finally, dietary personalization is closely integrated with preventive and therapeutic strategies across the lifespan. Indeed, dietary management should no more be considered a mere consequence of diagnosis but an active tool for both prevention and treatment. In fact, early-life dietary diversity and timely allergen introduction are associated with reduced food allergy risk, while intervention at younger ages is linked to a higher likelihood of durable tolerance induction (107, 108).

Collectively, integrating refined OFC strategies with tailored and socially informed dietary planning may support a transition toward a more patient-centred precision care in FA.

## 5 Challenges and limitations

Despite the significant advances and potential benefits offered by innovative approaches, several challenges and limitations may hamper their widespread clinical implementation, as summarised in Table 2.

Clinical readiness, which is the degree to which a diagnostic or therapeutic tool has been validated, standardized, and implemented for routine use in clinical practice varies across these techniques. It encompasses factors such as evidence of effectiveness, reproducibility, regulatory approval, required infrastructure, technical complexity, and accessibility across different healthcare settings. Understanding the clinical readiness of a given technique is essential for determining its applicability, guiding its integration into diagnostic workflows, and identifying which tools are suitable for secondary care, tertiary care, or remain restricted to research and specialized centres. In the context of FA, clinical readiness helps differentiate approaches that are theoretically fully implementable, such as CRD, from those that are partially validated/implementable or at present only investigational, including functional assays (BAT, MAT), IgG4/IgE ratios, epitopes-base testing and multi-omics strategies (10). One factor among other factors influencing clinical readiness, particularly reproducibility, of innovative techniques is heterogeneity across populations and allergens. Indeed, the performance of tests and interpretation may vary according to ethnicity, geography, age group, atopic background and prevalence of pollens sensitization (10).

The context-dependency is particularly relevant for CRD and AI with multi-omics approaches. CRD use is well validated and routinary used for peanut allergy and possibly sesame, while its predictive value is less established for others, such as lupin or other tree-nuts in different geographic regions (10, 12, 57). Machine-learning models elaborated in one pediatric cohort may not be directly transferable to another one. Also, IgG4/IgE ratios, which may reflect immunologic tolerance development, remains inconsistent across studies and patient populations (29, 59, 63).

CRD, cellular assays and epitope-specific IgE profiling have improved risk stratification and identification of persistent vs. transient allergies (29, 30, 36, 37, 79) yet widespread clinical implementation is limited by cost, technical complexity, and variability in assay standardization (12, 47, 48).

Standardisation issues are other relevant factors limiting clinical readiness and include, among others, source/composition of allergen extracts, antigen concentrations, gating strategy, marker choice, activation thresholds in flow cytometry-based assays, batch effects, platform to platform comparability and interlaboratory reproducibility (21, 56).

Functional assays, such as basophil and mast cell activation tests, offer improved predictive performance for clinical reactivity and response to immunotherapy (15–22, 48–52). However, they are partially validated (the BAT) or investigational (the MAT). These assays require specialized laboratory infrastructure, experienced personnel, and standardized protocols, limiting accessibility and reproducibility. In addition, their integration into routine clinical workflows is hampered by logistical and regulatory limitations. More specifically the MAT appears at present operationally unrealistic in many settings.

The advent of multi-omics approaches, including genomics, transcriptomics, proteomics, and metabolomics, coupled with AI-based predictive modelling, could allow precision FA management (109). These technologies enable the identification of early-life biomarkers, personalized risk profiles, and dynamic immune-profiling that could possibly guide therapeutic interventions in the future. Nevertheless, high costs, the need for large, ethnically diverse longitudinal cohorts, and data harmonization challenges immediate clinical translation. AI models risk overfitting or bias when trained on small or non-representative datasets, and their implementation requires clinician-friendly interfaces, training, and robust validation (30, 31, 110). Therefore, despite their considerable potential, these approaches remain at present far from routine clinical deployment. Key barriers include lack of standardization, high costs, limited reproducibility, and insufficient external validation.

Ethical and regulatory considerations also present barriers. Genomic and multi-omics data are inherently sensitive, raising concerns regarding privacy, informed consent, and equitable access. More precisely, ethical guidelines for clinical AI stress the importance of transparency, accountability, privacy, fairness, and careful testing before these tools are used in healthcare. In Europe, these principles are summarised in one official guidance, the European Ethical Framework for Clinical AI Systems 2025, which highlights that AI systems should support human decision-making, be understandable, and operate safely and fairly (111). In the United States, similar discussions have prompted the regulatory agencies such as the Food and Drug Administration (FDA), to release indications on the development and use of AI and machine learning in medical devices, highlighting the need for robust validation, transparency, and post-market monitoring (112). Besides, approval pathways for AI-driven diagnostic tools remain underdeveloped, complicating clinical adoption. Interdisciplinary collaboration among immunologists, paediatricians, bioinformaticians, and ethicists is essential to address these issues.

Moreover, heterogeneity in study design, allergen exposure, and immunotherapy protocols complicates data interpretation and meta-analyses, limiting generalisability. This limitation applies broadly to all innovative techniques in this field.

Long-term studies are needed to validate predictive biomarkers, refine CRD and epitope mapping approaches, and assess cost-effectiveness in real-world clinical settings. Overcoming these challenges is critical to move FA management from reactive care toward proactive, precision-guided prevention and treatment strategies.

Finally, disparities in access to diagnostic techniques-especially for costly innovative techniques, as well as for the overall management of FA, pose significant limitations to their routinary implementation. This disproportionately affect minority populations and low-income families, as highlighted in a recent AAAI position statement (113).

## 6 Future directions

The integration of advanced molecular diagnostics, multi-omics technologies, and AI has the potential to transform the investigation and management of FA (109). A clearer distinction among innovative techniques between what is currently applicable, what remains specialized, and what is still investigational is essential to guide clinical translation. More precisely, the near-term impact will derive from e optimized use of already available tools combined with the selective incorporation of advanced diagnostics in specialized settings.

At present, extract-based sIgE testing and SPT remain the cornerstone of diagnosis, with CRD and the BAT already providing added value in selected clinical scenarios, particularly for risk stratification and the interpretation of cross-reactivity. These tools are increasingly accessible and can be realistically implemented in many secondary and tertiary care settings. A second tier includes approaches that are close to broader clinical adoption but still largely confined to specialized centres. These include the MAT, epitope-resolved IgE profiling, and allergen-specific IgG4/IgE ratios, which offer important functional and prognostic insights, such as the ability to distinguish true clinical allergy from asymptomatic sensitization, predict severe reactions, response to OIT (29, 30, 59, 63). These techniques are limited by technical complexity, standardization issues, and restricted availability. In contrast, multi-omics platforms and AI-driven predictive models remain largely investigational.

By integrating molecular and functional assays with multi-omics and AI-based models, innovative techniques approaches can capture the full complexity of the disease, allowing for risk prediction during early-life, more accurate diagnosis, and personalized treatment strategies (109).

Over the next 3–5 years, key research priorities should include large, multicentre, longitudinal studies with standardized phenotyping to validate emerging biomarkers, direct comparisons of diagnostic tools in clinically relevant decision pathways, including their ability to reduce the need for OFCs, robust cost-effectiveness analyses and the development of regulatory and data-governance frameworks for AI-based diagnostics (109).

Overall, the future of FA management may progressively rely on the stepwise integration of molecular diagnostics, functional assays, multi-omics, and AI-based models, shifting care from a reactive, symptom-based paradigm toward a more proactive and personalized approach.
